# A Cue for Rational Reasoning: Introducing a Reference Point in Cognitive Reflection Tasks

**DOI:** 10.5964/ejop.v15i1.1701

**Published:** 2019-02-28

**Authors:** Kaja Damnjanović, Vera Novković, Irena Pavlović, Sandra Ilić, Slobodan Pantelić

**Affiliations:** a Laboratory of Experimental Psychology, Department of Psychology, Faculty of Philosophy, University of Belgrade, Belgrade, Serbia; University of Belgrade, Belgrade, Serbia

**Keywords:** dual processes, cognitive reflection test, ecological rationality, heuristics, a reference point

## Abstract

The dual process framework posits that we reason using the quick System 1, and the deliberate System 2, both of which are part of our “adaptive toolbox”. The Cognitive Reflection Test (CRT) estimates which system was used to solve a reasoning problem. Usually, the CRT tasks are solved incorrectly by using System 1, and correctly through System 2. We have applied the reference point hypothesis to the tasks of the CRT and proposed that this change would facilitate the switch between systems, resulting in better performance on the version of the test with a reference point, compared to the CRT without one. The results confirmed our assumptions, as evidenced by a generally higher score on the CRT with a reference point, albeit with different effects between items.

We are cooking a lovely soup, and we see our pot on the stove, fully realizing that the pot-handles are hot. However, when the soup starts to boil, immediately we reach for the pot-handles in order to remove the pot from the heat-source, even though we know we are making a mistake. And just like that, we get burned. Awareness of the mistake aside, in this situation, two response types were conflicted, and the automatic one prevailed over reasonable behavior^i^. But what would have been the outcome of this cognitive battle if there had been a kitchen cloth we could have turned to for aid between us and the pot?

The traditional notions of dual process theories (DP) posit that there are two distinct kinds of thought processes: heuristic and analytic (Evans, 1984; Stanovich & West, 2000; Wason & Evans, 1974-1975). Heuristic processes stem from the fast and intuitive System 1, and analytic processes stem from System 2, which is slow, deliberate and stepwise. Systematic deviations from reasoning norms are thought to be rooted in the heuristic mind, which is sometimes prone to errors, because it tends to ignore a part of the available information. Conceptual and applicative limitations of the DP framework are the subjects of scholarly debates (e.g. Evans & Stanovich, 2013); still, all of the iterations of the model of the duality of cognitive processing share two stances about our mind’s features: a) there are two subsystems within our cognitive system, and b) they have different phenomenology. While they are well elaborated in theoretical models, measuring heuristic processes is still indirect. One of the techniques used is the Cognitive Reflection Test (CRT) (Frederick, 2005; Toplak, West, & Stanovich, 2014), devised to identify which system a person has used while solving a particular task. Namely, in order to produce a correct answer, participants would have to effectively monitor *and* correct their impulsive, heuristic-response tendencies, as well as engaging in further reflection (Frederick, 2005; Primi, Morsanyi, Chiesi, Donati, & Hamilton, 2016), and this switch between the System 1 and System 2 has to happen (Pennycook, 2018). The test originally contained three items, but it has been developed and expanded throughout the years, in an effort to create a measure which would not be easily skewed by either educational or developmental differences between subjects (Primi et al., 2016). Results indicate that the prolonged versions all present an adequate measure of the ability to switch between System 1 and System 2 processing, while also achieving the levels of between-subject distinction required (Primi et al., 2016; Toplak et al., 2014).

The link between System 1 and System 2, as well as the nature of that relationship is currently the “it” question in the field of dual process theories. This issue can be approached from two positions, the first one being that there is a conflict between the two systems (Evans, 2006; Evans & Stanovich, 2013; Fontinha & Mascarenhas, 2017). However, the idea of conflict automatically implies that System 2 was autonomously activated from the start, by a stimulus (task). This violates the basic assumption of the dual process theories, which posits that System 2 is *deliberately*, not autonomously, activated. The second approach, and the one that we employ, proposes that the relationship between the two systems can be defined as a switch occurring between them (Evans & Stanovich, 2013; Pennycook, 2018; Pennycook, Fugelsang, & Koehler, 2015). The main reason behind the assertion that the switch does, in fact, happen lies within the finding that incorrect answers on CRT are *almost never* random (Primi et al., 2016). Namely, while the participants’ answers can be either correct or wrong, the wrong answers CRT items facilitate are, to some extent, predictable - that is, when participants make a mistake, a large percentage of the incorrect answers can be clustered as "typically erroneous”, and presumably stemming from heuristics.

The cause for the switch between the employed systems can be something as simple as an instruction (Evans, 2010), but it can also be prompted by the high level of importance of the situation, like in speed or strength testing. However, in most situations where we *do* employ System 2 reasoning, the cues are not so obvious, and there is no blatant instruction to “use your head” (Pennycook, 2018). So, why does the switch happen?

Based on the concept of a reference point (RP), initially introduced in the prospect theory (Kahneman & Tversky, 1979; Tversky & Kahneman, 1992), the presence of a reference point within a situation could shift our thinking from one specific pattern of cognitive processing to another. The prospect theory postulates the concept of the RP as the demarcation between the two zones (gain and loss) in the value function. The two zones were initially proposed by Markowitz (1952), but he does not discuss the transition from one zone to another. The implication of distinguishing these two zones is the assumption about the differential psychological treatment of gains and losses: risk-taking and risk aversion, or in more general terms, psychophysical function. The idea of the RP serves as the explanation for the mechanisms of some cognitive illusions, such as the framing effect. The RP directs the selection of the aspects of the situation that will be in focus, which consequently shapes information processing. For example, what the participants are going to do with two glasses, empty and full, depends on the reference point masked within the instruction (Sher & McKenzie, 2006).

Applying it to the field of judgement, McKenzie and Nelson (2003) argued that if a reference point is not presented, it is implicitly inferred from the way the information is presented. The reference point hypothesis (RPH) is the foundation on which the information leakage approach was built (ILA; Sher & McKenzie, 2006). The ILA posits that the way in which information is presented and conveyed to subjects influences the resulting inferences from said information. In other words, two logically equivalent sentences (e.g. a sentence in active and a sentence in passive voice) can be information non-equivalent, when additional information, stemming from the choice of the form of the sentence, leaks out above and beyond the literal presented information. While this implicit information can be misleading or used for manipulation, it can also facilitate successful communication and information transfer. Applied to reasoning processes, this means that if a reference point is given within or can be easily extracted from the presented information, it might influence our ability to shift from System 1 to System 2 reasoning. This notion is in line with the concept of ecological rationality (ER; Gigerenzer, 2008; Gigerenzer & Brighton, 2009). According to ER, people pursue objectives in their environments and they do so by utilizing their adaptive toolbox, which is not *normatively* but *ecologically* rational. That ecological rationality is defined functionally, as correspondence and congruency between the utilization of specific tools from the toolbox and the context in which they are used, and this congruency is triggered by environmental cues. Human reasoning is in constant interaction with the environment and uses cues in order to be adaptive. If, for instance, an employed heuristic proves to be adaptive, it is also, from the perspective of ER, considered to be rational, that is - congruent with the structure of the environment, which is the consequence of the reaction to a particular signal from the environment. Presumably, and contrary to this, a person can also use a cue from the environment in the opposite sense, as a signal to override the aforementioned tendency to ignore a part of the information, and engage in further reflection, i.e. switch to System 2.

We propose that this presumption can be applied to reasoning tasks. If a person is presented with two equivalent tasks, one of which contains the cue to engage in System 2 reasoning, the person will extract additional information from this cue, and have a better chance of giving the correct answer. Low rates of correct answers on the CRT are sometimes attributed to the situation being wrongly interpreted as if it yields no cognitive conflict, leading the participant to give the first answer that comes to his or her mind, which is also incorrect (Fontinha & Mascarenhas, 2017). In order to solve a problem, people have to be able to notice the problem, but they also need to pay attention to the problem’s premises (Mata, Ferreira, Voss, & Kollei, 2017). Previous research indeed shows that participants score higher on reasoning tasks when the conflict has been removed from the presented information (De Neys & Glumicic, 2008; Ferreira, Mata, Donkin, Sherman, & Ihmels, 2016; Mata & Almeida, 2014; Mata, Ferreira, & Sherman, 2013; Pennycook, Fugelsang, & Koehler, 2015). Another way to explain this is by using the concept of “cognitive miserliness” (Stupple, Pitchford, Ball, Hunt, & Steel, 2017; Toplak, West, & Stanovich, 2011), which posits that participants, when completing the reasoning tasks, don’t even try to pay attention to all of the presented information. Instead, they just give the first answer that comes into their minds. In other words, even though the participants might have been aware of the possibility that there was a conflict and that their answer was wrong, they nevertheless simply chose to give the intuitive satisficing response, which is in line with the finding that participants who gave the wrong answers also estimated their confidence about their answers as lower (De Neys, Rossi, & Houdé, 2013; Stupple et al., 2017), which could go along with the notion that the participants are aware of the level of their answers’ accuracy. However, for some types of reasoning tasks, such as syllogistic reasoning, or base rate, the results are almost straightforward: the participants are not good judges of their own performance (Bajšanski, Močibob, & Valerjev, 2014; Dujmović & Valerjev, 2018; Thompson & Johnson, 2014; Thompson & Morsanyi, 2012). Furthermore, some studies show that the first intuitive answer that comes to mind is usually accompanied by a very strong feeling of rightness, which in turn determines the probability of engaging in System 2 processing (Thompson & Morsanyi, 2012). With regard to the differences in reaction time, in the two-response paradigm, in which participants give their first intuitive response, evaluate how right the response feels, and then give the second response, the first response is faster and given heuristically (System 1). The second response comes after the analytical System 2 has been deployed, either through environmental cues or directly by the participants’ conflict detection (Bago & De Neys, 2017; Dujmović & Valerjev, 2018; Pennycook, 2018). The process of “switching” from System 1 to System 2 is, aside from rendering confidence estimation lower, also expected to take more time. Therefore, the inherent feature of System 2 processing is its slowness, while the heuristic processes and their respective answers, being intuitive, just “pop up” and require less time (Alós-Ferrer, Garagnani, & Hügelschäfer, 2016). We should note that the response time asymmetry, usually used as the observable *differentia specifica* of the two processing types, is not without doubt, as discussed by many researchers (Ball, Thompson, & Stupple, 2018; Dujmović & Valerjev, 2018; Stupple, Ball, Evans, & Kamal-Smith, 2011; Trippas, Thompson, & Handley, 2017). One of the possible (and used) explanations is that the prolonged response times (e.g. Ball, Thompson, & Stupple, 2018), which accompany heuristic responses in the original trials, compared to responses on items with an RP, are more appropriately explained as an effect of conflict detection and resolution, which probably mainly occurs without conscious effort. Second, the reliability of response time as an indicator of the two types of processing is probably in interaction with the varying logical abilities of the participants.

Based on the concepts of ecological rationality, and the model of information leakage, we suggest that adding a reference point to the CRT problem solving tasks might serve as an environmental cue for analytical reasoning. The goal of this research was to reveal whether the addition of a reference point (RP) has an effect on inducing further reflection in cognitive reflection tasks, as well as to pinpoint exactly in which cases the RP addition facilitated the proverbial "shift" to System 2, i.e. what kind of RP prompts us to devote more cognitive resources to a certain task. We hypothesized that: a) adding the reference point to the CRT tasks would increase the average number of correct answers, while it would decrease the number of heuristic answers, compared to the standard version of the CRT without an added reference point; b) the reaction time would be shorter for standard tasks than for the tasks with a reference point (Alós-Ferrer et al., 2016); c) the standard tasks would be accompanied by a higher estimation on the metacognitive self-assessment scale, while the self-confidence score would be lower on the tasks containing a reference point (Primi et al., 2016; Thompson & Morsanyi, 2012).

## Method

### Sample

The participants were first-year students of the University of Belgrade, Faculty of Philosophy, Department of Psychology (*N* = 94, 77.66% female; the average age 20), who received course credit for completing the experiment.

### Materials

The stimuli were reasoning tasks compiled from three versions of the Cognitive Reflection Test. These are tasks in which the correct answer can be easily calculated with basic algebra skills, but are constructed in such a way that a typical wrong answer appears to the participants as the correct one. Two parallel versions of each of the tasks, like the two versions of the CRT, were used: one was the standard (CRTs), and the other was an adjusted version, dubbed the CRTr (Damnjanović & Teovanović, 2017). The conventional CRT was created by combining all three standard versions of the test: the original three-item CRT (Frederick, 2005), the 7-item CRT (Primi et al., 2016) and the 9-item CRT (Toplak et al., 2014). In the construction of the tasks in the CRTr, every item of the CRTs underwent additive changes; that is, a reference point was specified and introduced to each task, while the formal aspects of the tasks were kept constant. The number of words, characters, and syllables of the items did not differ by more than 10% across the tasks for a given pair. For example, at the very beginning of the question “A man buys a pig for $60, sells it for $70…” the clause “A man has $80.” was added as a starting point for further calculation (the list of stimuli is given in Appendix). However, this addition of the RP is not indisputable. While the most straightforward operationalization of the theoretical concept of the RP was in the ‘pig-salesman’ task, changes in other items were not so straightforward. These specific challenges stem from the fact that the items of CRTs are not uniform regarding the operation (e.g. subtraction, speed comparison…) which they require for successful solving. The rationale for using these stimuli was based on intersecting two criteria. First, the item had to be an item from any of the validated CRTs, which means that it could yield both normatively correct and typically incorrect answers. The second was the idea that an RP could either be added before any calculus was needed (e.g. the pig tasks), or that it could focus the participant on the aspects of the tasks which were previously masked by the existing conflict, either by clarifying the offered information (e.g. printer), or by swapping the subject and the object (e.g. Marko’s grade).

In both versions, one dummy question was added, which had the structure of a simple string of calculations, without a conflicting aspect, with the aim of nesting of the participants more firmly into the mode of task solving. Both versions were tested in a previous study in pen-and-paper form (Damnjanović & Teovanović, 2017).

For both versions of every task, three types of answers were coded: correct (mathematically), heuristic (typical erroneous), and atypical - all other answers that were not correct mathematically *and* weren’t typical heuristic answers either.

Metacognitive self-assessment was conducted using a Likert-type 7-point scale on which the participants answered the question “How confident are you of your answer?”.

### Design

In a counterbalanced repeated design, the participants were randomly assigned to one of the two experimental groups (CRTs or CRTr), so that one group first completed the standard version of the test (CRTs), and then two weeks later completed the CRTr version, while the other group solved the tests in reverse order.

The independent variable had two levels (CRTs, CRTr) – whether an item had a reference point included or not. The dependent variables were: the number of correct responses (range from 0 to 8), the number of heuristic responses (0-8), response time from the moment when the item text appeared on the screen to when the participant gave an answer, and confidence – the participants’ estimate of how confident they were of the answer they gave (ranging from 1 to 7).

### Procedure

The experiment was constructed in OpenSesame v.2.9, and it consisted of three exercise tasks and nine main tasks, of which one was the dummy question, whose answers did not count. They were administered in random order to 94 participants. No time limit was imposed, but the participants were asked to respond “as quickly and as correctly” as they could in the instruction preceding the experiment. The registered response time for every item ranged from 23 to 104 seconds (*M* = 46.24). Every item was followed by a 7-point self-confidence scale as a measure of metacognitive self-assessment. Prior to the main part of the experiment, the participants went through a short trial, composed of three items resembling the CRT ones. The data from the exercise was not used in the analyses. The study was conducted during the year 2017 in four sessions, in groups of about 25 subjects. Prior to stimuli presentation, the participants signed a written consent form and were given instructions in both written and oral form.

## Results

### Scores on the CRTr and CRTs

In order to test whether there was an effect of the order of presentation of the two versions of the test (CRTs and CRTr) a two-way ANOVA was conducted. The order of presentation (two levels: session one and session two) and the version of the CRT (two levels: CRTs and CRTr) were used as the independent variables. The total number of tasks solved correctly by each participant was used as the dependent variable. The interaction between the two factors wasn’t significant, *F*(1, 327) = 0.076, *p* = .783. The main effect of the order was not significant either, *F*(1, 327) = 0.184, *p* = .668, while the main effect of the version of the test was significant, *F*(1, 327) = 19.259, *p* < .001, η^2^ = .056. We have also analyzed the total number of heuristic answers per participant as the dependent variable. The independent variables in this two-way ANOVA were again the order of presentation and the version of the test. The analysis yielded results that confirm the absence of the effect of the order of presentation. Namely the only significant main effect was the effect of the version of the test, *F*(1, 327) = 33.726, *p* < .001, η^2^ = .093. The main effect of the order of presentation as well as the interaction between the two factors were not statistically significant (*p* = .355 and *p* = .621, respectively). In short, the analysis showed that the participants’ solving of the tasks was not influenced by the order of presentation, but by the version of the test they were presented with.

In order to test the differences in the numbers of correct, heuristic and atypical answers given in the two versions of the test, the total correct, heuristic and atypical scores for two versions of the test were used as the dependent variables. Mean total scores and standard deviations for each type of answer, as well as the mean response time per item and mean confidence level for both experimental situations are shown in Table 1.

**Table 1:** Means and Standard Deviations for Total Scores, Response Time and Confidence Level on Standard (CRTs) and the Version With Reference Point (CRTr)

Test Version	*n*	Response type						Response time		Confidence	
		Correct		Heuristic		Atypical					
		*M*	*SD*	*M*	*SD*	*M*	*SD*	*M*	*SD*	*M*	*SD*
CRTs	90	3.66	2.01	2.76	1.51	1.58	1.24	45.45	14.23	4.11	1.27
CRTr	85	4.61	1.99	1.78	1.30	1.61	1.32	47.27	14.69	4.21	1.24

*Note.* Means (*M*) and standard deviations (*SD*) on total correct, heuristic, and atypical scores, response time in seconds, and self-confidence for each test version separately.

**Figure 1:**
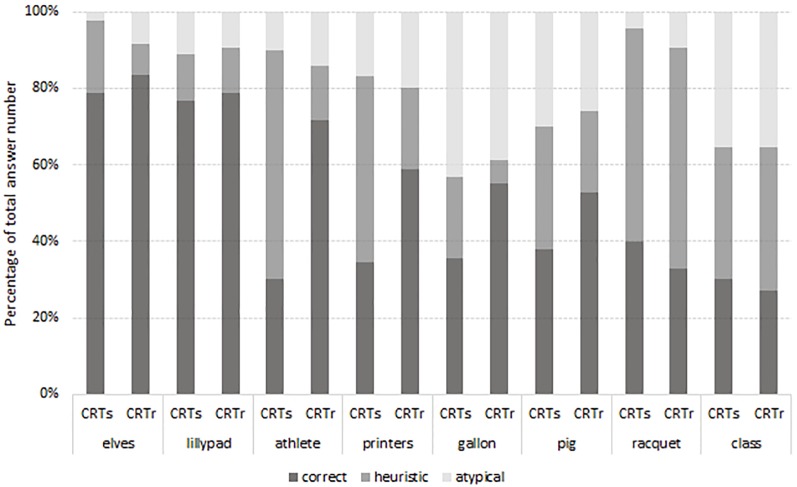
Percentages of correct, heuristic, and atypical answers per both versions of each task.

The number of correct answers significantly differed between the CRTs, 99% CI [3.09, 4.17], and the CRTr, 99% CI [4.05, 5.17], *t*(84) = -3.24, *p* < .01. Average number of heuristic answers also differed between the CRTs, 99% CI [2.41, 3.26], and the CRTr, CI [1.41, 2.14], *t*(84) = 4.705, *p* < .001. The distribution of answer types per each task, between the two test versions, can be seen in Figure 1. No significant difference was registered in the average number of atypical, *t*(84) = -0.182, *p* = .856, responses, nor in the response time, *t*(84) = -0.970, *p* = .335, and confidence self-assessment, *t*(84) = -0.510, *p* = .611, between the two test versions, not even when explored per each *task*.

#### Scores on Pairs of Tasks

Further analysis focused on the correct, and heuristic answers, since atypical responses were non-indicative in the applied theoretical framework. Each stimulus yielded either the correct or the heuristic answer, and the two versions of the same task, with the percentage comparison of both correct and heuristic answers for the two test versions are shown in Table 2. In order to test whether there were more correct responses on the CRTr than the CRTs, a t-test analysis was conducted, with the task version as the grouping factor. The dependent variable was computed as follows: the correct answers were coded as 1, and all the other answers (both heuristic and atypical) were coded as 0.

**Table 2:** Differences in the Proportion of Correct and Heuristic Answers for Two Test Versions per Stimulus

Test Version	Correct					Heuristic				
	P	95% CI		*t*(84)	Cohen’s *d*	P	95% CI		*t*(84)	Cohen’s *d*
		*LL*	*UL*				*LL*	*UL*		
class				0.491	-0.895				-0.466	1.076
CRTs	30.59%	20.73	40.44			34.12%	23.98	44.26		
CRTr	27.06%	17.56	36.56			37.65%	27.29	48.01		
athlete				-5.318***	4.323				6.843***	10.028
CRTs	31.76%	21.81	31.72			57.65%	47.08	68.21		
CRTr	71.76%	62.14	81.39			14.12%	6.67	21.56		
gallon				-2.547**	-6.544				2.788**	0
CRTs	36.47%	26.18	46.76			20.00%	11.45	28.55		
CRTr	55.29%	44.66	65.93			5.88%	0.85	10.91		
lily pad				-0.652	-0.233				0.257	0.150
CRTs	75.29%	66.07	84.52			12.94%	5.76	20.12		
CRTr	78.82%	70.09	87.56			11.76%	4.87	18.65		
elves				-0.686	-0.293				2.185*	0.764
CRTs	80.00%	71.45	88.55			17.65%	9.49	25.80		
CRTr	83.53%	75.60	91.46			8.24%	2.36	14.11		
racquet				0.948	-10.063				-0.307	7.387
CRTs	40.00%	29.52	50.48			55.29%	44.66	65.93		
CRTr	32.94%	22.89	42.99			57.65%	47.08	68.21		
printers				-3.256**	-5.218				3.534***	-4.518
CRTs	35.29%	25.07	45.51			47.06%	36.38	57.73		
CRTr	58.82%	48.30	69.35			21.18%	12.44	29.91		
pig				-2.155*	11.594				1.533	-3.747
CRTs	36.47%	26.18	46.76			31.76%	21.81	41.72		
CRTr	52.94%	42.27	63.62			21.18%	12.44	29.91		

*Note.* CRTs = tasks without reference point; CRTr = tasks with reference point; P = percentage of correct/heuristic answers in comparison to total answers per item. CI = confidence interval; *LL* = lower limit; *UL* = upper limit.. *t*(df) = value and degrees of freedom.

*p < .05. **p < .01. ***p < .001.

A significant difference in the proportion of correct answers between the two item versions was registered on four pairs of tasks (*athlete, gallon, printers*, and *pig*): the items with a reference point produced more correct responses than their CRTs versions (*t* statistics ranging from -5.318 to -2.155). On the rest of the task pairs, there were no differences between items. The *t*-test analysis was performed on the heuristic answers as well, and the dependent variable was computed so that heuristic answers were coded as 1, and all the other answers (both normative and atypical) were coded as 0. Again, a significant difference in the proportion of heuristic answers between the two item versions was registered on four pairs of tasks (*athlete, gallon, printers*, and *elves*). The items with a reference point, in total, did produce fewer heuristic responses than their CRTs counterparts (*t* statistics for items that differed significantly ranging from 2.185 to 6.843).

To sum it up, the reference point inclusion both increased the number of normatively correct answers *and* reduced the number of heuristic answers on three tasks: *athlete*, *gallon*, and *printers*. On two items, the manipulation was successful only partially; that is, the added reference point influenced one of the types of answers. On the item *pig*, the added reference point increased the correctness, but did not decrease the number of heuristic answers, and on the item *elves* the RP did not increase correctness but did decrease the number of heuristic answers. The rest of the items (*class, lily pad*, and *racquet*) were not significantly affected by the introduction of the reference point.

#### Response Time and Self-Assessed Confidence

A correlation between response time and self-assessed confidence has been registered on both the CRTs, *r*(718) = .53, *p* < .001, and the CRTr, *r*(678) = .28, *p* < .001, version of the test. In order to test whether response time depended on the task type, or the status of the answer, a repeated measures ANOVA was conducted. The status of the answer (correct, heuristic, and atypical) and the version of CRT (CRTs, CRTr) were used as the factors and RT as the dependent variable. The analysis showed no significant interaction between the factors, *F*(2, 112) = 0.222, *p* = .801, as well as no significant main effects of either the status of the answer, *F*(2, 112) = 0.251, *p* = .779, nor the version of the CRT, *F*(1, 112) = 1.599, *p* = .209. The same analysis was conducted to observe the relationship between the status of the answer and the version of the CRT with self-assessed confidence measures. A repeated-measures ANOVA showed no significant interaction, *F*(2, 112) = 2.134, *p* = .123, and no significant effect of the version of the CRT on self-assessed confidence, *F*(1, 66) = 3.026, *p* = .087. A significant effect of the status of the answer has been detected, *F*(2, 66) = 31.331, *p* < .001, η^2^ = 0.487. Post-hoc analyses using the Bonferroni post-hoc criterion for significance showed that all the levels of the status of the answer factor differed significantly (*p* values ranging from < .001 to .01). The participants reported the highest confidence when giving correct answers, *M* = 4.718, *SD* = 1.200. When the participants gave heuristic answers, their self-assessed confidence level was in the middle, *M* = 3.677, *SD* = 1.622, whilst the lowest assessment occured when giving atypical answers, *M* = 2.859, *SD* = 1.668.

## Discussion

Our study was conducted with the aim of examining the effect of introducing a reference point into conflict problem-solving tasks, in order to trigger a rational approach to the same task. This is something of a “hot topic” in the research-grounded dual-process approach, and an extensive number of studies in the field currently aim to provide an answer to the question: “What could make us think (and therefore act) *rationally,* not quickly and *heuristically*, in situations which cause conflicting cognitive responses?” (Evans & Stanovich, 2013; Pennycook, 2018; Primi et al., 2016). On one end of the imaginary continuum of answers to this question is the notion that the human *ratio* is inherently “flawed” and hence systematically biased, or irrational – as postulated in more formal models, *e.g*. prospect theory (Kahneman & Tversky, 1979; Tversky & Kahneman, 1992) - while on the opposite end is the idea that environmental cues can improve (ecological) rationality (Gigerenzer, 2008). In accordance with our aim, we applied the latter to the infamous Cognitive Reflection Test and the different types of answers which its conflict-invoking tasks can yield. We added a reference point to its tasks, which was meant to act as a helping *cue* from the environment, inciting a change in our reasoning, by using the tools from our so-called “adaptive toolbox”. Basically, it would mean that cued answers would require more time, but be correct more often, and score lower on metacognitive self-assessments. We have tested this by comparing scores from the two versions of the CRT, the original one and a version whose tasks contained cues (that is, reference points).

Our assumptions were confirmed on several of the tasks, though not on all of them. The stimuli that proved to be in line with our expectations regarding the proportions of correct and heuristic answers after the manipulation are ‘athlete’, ‘printer’, and ‘gallon’ tasks. The ‘pig-salesman’ and the ‘elves’ tasks also confirm our hypotheses, but not in their entirety. That is to say, when the participants were solving the first three tasks mentioned, they were more correct when a reference point was given, *and* also gave fewer heuristic answers. However, while the ‘pig-salesman’ task with an RP did produce a spike in correct answers, compared to its conventional counterpart, the reference point did not make a difference in the prevalence of heuristic answers. The opposite is true of the ‘elves’ task: our cue did not cause the participants to be “smarter” about solving it, seeing as how the number of correct answers is the same on both tests, but it did seem to stop them from latching onto the first seemingly feasible answer, since not as many of their answers were heuristic. So what makes these five stimuli special? The reasonable explanation would suggest that the reference point presented within them was particularly effective (Bago & De Neys, 2017; Pennycook, 2018). However, when their reference points are compared, the similarities between RPs can be found in pairs: RPs based on fractions calculus (‘athlete’ and ‘gallon’), RPs based on indirect comparison of speed (‘printers’ and ‘elves’), or on the time necessary to perform a specific task. An RP in its most literal sense is the one introduced to the ‘pig-salesman’ task: a starting point for simple addition and subtraction operations. The task ‘printers’ with a reference point is the *second*-most successful of them all (the ‘athletes’ task being the first), even though it contains a double reference point (which might have over-simplified it) although some participants still found it more difficult than a fraction-based task. Further research is required to refine these findings and unearth the specifics of the “perfect” RP.

A second cluster of stimuli has proven impervious to our experimental manipulation, consisting of the ‘lily pad’, ‘racquet’, and ‘class’ stimuli. Firstly, the ‘lily pad’ task was overwhelmingly easy for all the participants in both versions of the test. Additionally, the lack of difference in correct-answer proportions between the versions reveals that the RPs in the two versions were not obviously (or at all) different to the participants. A similar explanation might be valid for the racquet task, the ‘paradigm’ CRT task, which is difficult in any of its forms: a more obvious and simplifying RP might be required to cause an effect. The unaffected 'class' and 'racquet' stimuli have different kinds of RPs from the five tasks which *were* affected, in the sense that their RPs offer different types of information, and require a unique type of calculation compared to the other tasks in the CRT. The RP in the ‘class’ and ‘racquet’ tasks contained no additional numbers to use in the calculation: rather, the c(l)ue referred to how the different elements in the task were related. It could be that these cues weren’t carrying enough information to trigger analytical thinking, as well as that the RP wasn’t eye-catching enough for the participants, so they focused on the same aspects in both versions of the test. In order to solve a problem, people need not only be able to solve it, but to pay attention to the problem’s premises (Mata et al., 2017), and a lack of this might be the case with the ‘class’ and ‘racquet’ tasks.

Considering the potential effect of the reference point on response time, we assumed that the engagement in System 2 processing would go hand-in-hand with an RT extension, since deliberation takes more time than “jumping the gun” and answering heuristically (Alós-Ferrer et al., 2016). However, there were no overall differences in RT between the two versions of the *tasks*, nor between the different types of *answers*. This absence of the anticipated difference in response time between heuristic and correct answers can be explained by the fact that the prolonged response times which accompanied heuristic responses in the tasks are probably an effect of conflict detection and resolution, which probably mainly occurs without conscious effort (Ball, Thompson, & Stupple, 2018). Secondly, speed asymmetry as an indicator of the differences between the two types of processing has been called into question (Ball, Thompson, & Stupple, 2018; De Neys, 2014; Dujmović & Valerjev, 2018; Evans, 2017; Stupple et al., 2011; Trippas, Thompson, & Handley, 2017) and the reliability of response time as the indicator of the two types of processing is probably in interaction with the varying logical abilities of the participants (Stupple et al., 2011), which were not included in the scope of this study. The situation is partly similar with the lack of certainty we intended to cause in our participants by the type of task, with no difference in confidence registered between those. On the other hand, the participants were more confident about the correctness of their solution when that solution was actually correct, which is in accordance with some previous results (De Neys et al., 2013; Thompson & Morsanyi, 2012). In fact, they were less confident when giving the wrong, heuristic answer, and the least confident when they gave atypical answers. This is in line with the aforementioned concept of "cognitive miserliness” (Stupple et al., 2017; Toplak et al., 2011), according to which the participants might be, when completing the tasks, aware of the possibility that there is a conflict and that their answer could be wrong, but nevertheless, they choose to give the first, intuitive and satisficing response, or a random wrong response, and thus estimate their confidence in their answers as lower (De Neys et al., 2013; Stupple et al., 2017).

These results confirm that reasoning doesn’t only depend on one’s cognitive ability, but also on the way (conflicting) information is presented (Fontinha & Mascarenhas, 2017). Participants do score higher on reasoning tasks when the information is presented without conflicts, as confirmed by an abundance of earlier research (De Neys & Glumicic, 2008; Ferreira et al., 2016; Mata & Almeida, 2014; Mata et al., 2013; Pennycook et al., 2015). The difference in this study is that we introduced a reference point which could help participants solve the problem *in spite of* the conflicting information, without changing the deep structure of the task, the calculations required for the correct answer, or the correct answer itself. This allowed better control in comparisons between the two forms of the tasks.

However, it should be noted that the distribution of the three types of answers was in contrast with the findings in the previous studies which employed CRT, in which the majority of answers were erroneous, mostly heuristic (Frederick, 2005; Primi et al., 2016; Toplak et al., 2014). In our data, the percentage of correct answers was usually the highest, due to the successful experimental manipulation, but also presumably due to the student sample, which certainly limits the degree of possible generalization. Furthermore, response time as the indicator of the type of processing is, as previously stated, a problematic measure, in both technical (the starting point for measurement) and interpretative manner, so these results should be taken cautiously into consideration (Stupple, Ball, Evans, & Kamal-Smith, 2011).

Finally, the RPs are still a work in progress. As is the usual problem with stimuli in the higher cognition paradigm (*e.g*. with the amount of information), it is a rather categorical and not a continuous measure. The RPs were not all of the same variety, and this is especially challenging, because tasks in the CRTs are not uniform in the calculus operations they require (and thus whether System 2 is in the game). One of the ways this could be remedied is by splitting the CRT into calculation-specific blocks, so that a comparison could be made between the types of stimuli and the types of their respective RPs. We do consider this lack of refinement to be a drawback in our study. This is particularly obvious with the *printers* and *athletes* tasks, which all the participants found to be very easy, but although they differ by type (technically, *printers* has a double reference point, and *athletes*’ RP is fraction-based) their reference points differ by many parameters, which were not strictly defined enough for us to pinpoint the exact specifics which swayed the difficulty one way or the other. The same goes for the racquet task – it stands to reason that addition and subtraction tasks should be easier than multiplication and fractioning, but the racquet task remains the most difficult in the CRT to solve, with or without an RP. Therefore, one of the main pitfalls in our research is this lack of foresight to have defined the RP by several parameters (*e.g.* calculations required, number of RPs, position of RP in the task), and then formulating the CRTr in accordance with those parameters, which would have perhaps allowed us to give a more precise answer to the “switch-inducing RP” question.

All the findings above seem to point in the same direction: the RP hypothesis *does have merit*, but the way we chose to test its validity needs to be refined. In accordance with ILA, the framing of the tasks *did* influence the way they were solved, which is also in line with ecological rationality, positing that human reasoning is influenced by cues from the environment. In our research, this environment was the reasoning task, and the cue that caused the participants to change their answers was the RP. When analyzed separately, these cues differed regarding both their formal aspects and their effectiveness. Further research should focus on how to make these cues more balanced in order to make them more effective, and more importantly, to help us better understand the interplay between the two types of processing.

## Appendix - Tasks

**Tasks of CRT without a reference point**
1. A man bought a pig for 60$, then sold it for 70$, then bought it again for 80$, and finally sold it again for 90$. How much did the man earn?
2. Marko got the fifteenth highest and, at the same time, fifteenth lowest grade in the whole class. How many students are there in this class?
3. If three elves can pack three toys in one hour, how many elves are needed to pack 6 toys in two hours?
4. It takes 5 minutes for 5 machines to print 5 posters. How much time is needed for 100 machines to print 100 posters?
5. In an athlete club, tall members have three times higher probability to win a medal than short members. This year, the club has won 60 medals until now. How many of these medals were won by short athletes?
6. A tennis racket and ball cost 1100 dinars together. The racket is 1000 dinars more expensive than the ball. How much is the ball?
7. If Ivan drinks one gallon of water in 6 days, and Marija drinks one gallon in 12 days, how many days do they need to drink one gallon of water together?
8. There’s a field of water lilies in a lake. The surface of the field is doubled every day. If it takes 48 days for the field of water lilies to cover the whole lake, how many days are required for the water lilies to cover half the lake?

**Tasks of CRT with a reference point**
1. A man has 80$ in his pocket. He bought a pig for 60$, then sold it for 70$, then bought it again for 80$, and finally sold it again for 90$. How much did the man earn?
2. A single grade that Marko got is, at the same time, the 15th highest and the 15th lowest grade in the whole class. How many students are there in this class?
3. If three elves can pack three toys in one hour, and one elf can pack one toy in one hour, how many elves are needed to pack 6 toys in two hours?
4. In 5 minutes, one machine prints out one poster. In 5 minutes, eight machines print out eight posters. How much time is needed for 100 machines to print 100 posters?
5. In an athlete club, tall members have three times higher probability to win a medal than short members; in other words the short members win every fourth medal. This year, the club has won 60 medals until now. How many of these medals were won by short athletes?
6. A tennis racket and ball cost 1100 dinars together. One of these things is a lot more expensive than the other: the ball is cheaper than the racket, by a 1000 dinars. How much is the ball?
7. If Ivan drinks one sixth (1/6) of a gallon of water in one day, and Marija drinks one twelfth (1/12) of a gallon in one day, how many days do they need to drink one gallon of water together?
8. There’s a field of water lilies in a lake. The surface of the field is doubled every day. If it takes 48 days for the field of water lilies to cover the whole lake, after how many days was half the lake covered in water lilies?

**Dummy question**

A pair of size 32 shoes costs 3000 dinars. Size 33 of the same model costs 3200 dinars, and size 34 costs 3400 dinars. Size 35 costs 3500, and size 37 costs 3600. How much is a size 38?
